# Microstructure Optimization for Design of Porous Tantalum Scaffolds Based on Mechanical Properties and Permeability

**DOI:** 10.3390/ma16247568

**Published:** 2023-12-08

**Authors:** Yikai Wang, Xiao Qin, Naixin Lv, Lin Gao, Changning Sun, Zhiqiang Tong, Dichen Li

**Affiliations:** 1 State Key Laboratory for Manufacturing System Engineering, School of Mechanical Engineering, Xi’an Jiaotong University, Xi’an 710054, China; wangyikai@stu.xjtu.edu.cn (Y.W.); qinxiao@stu.xjtu.edu.cn (X.Q.); lnx9228@stu.xjtu.edu.cn (N.L.); zhiqiang.tong@xjtu.edu.cn (Z.T.); dcli@mail.xjtu.edu.cn (D.L.); 2 National Medical Products Administration (NMPA), Key Laboratory for Research and Evaluation of Additive Manufacturing Medical Devices, Xi’an Jiaotong University, Xi’an 710054, China

**Keywords:** porous Ta, microstructure optimization, mechanical properties, permeability

## Abstract

Porous tantalum (Ta) implants have important clinical application prospects due to their appropriate elastic modulus, and their excellent bone growth and bone conduction ability. However, porous Ta microstructure designs generally mimic titanium (Ti) implants commonly used in the clinic, and there is a lack of research on the influence of the microstructure on the mechanical properties and penetration characteristics, which will greatly affect bone integration performance. This study explored the effects of different microstructure parameters, including the fillet radius of the middle plane and top planes, on the mechanics and permeability properties of porous Ta diamond cells through simulation, and put forward an optimization design with a 0.5 mm midplane fillet radius and 0.3 mm top-plane fillet radius in order to significantly decrease the stress concentration effect and improve permeability. On this basis, the porous Ta structures were prepared by Laser Powder Bed Fusion (LPBF) technology and evaluated before and after microstructural optimization. The elastic modulus and the yield strength were increased by 2.31% and 10.39%, respectively. At the same time, the permeability of the optimized structure was also increased by 8.25%. The optimized microstructure design of porous Ta has important medical application value.

## 1. Introduction

Tantalum and its compounds have been widely used in aerospace, electronics, and especially medical fields due to their excellent properties such as high hardness, high toughness, high ductility, and corrosion resistance [1,2,3]. Porous Ta is generally considered a promising implantable biomaterial due to its biocompatibility, non-cytotoxicity, and suitability for the attachment and growth of osteoblasts [1,4]. In recent years, more and more clinical orthopedic implants made of porous Ta materials have been used, including artificial humerus femoral prostheses [5], ankle and hip prostheses [6] and acetabular pelvic prostheses [7]. In addition, compared with other porous metals, the friction coefficient between porous Ta implants and human bone is 40~80% higher [8,9], which is conducive to its binding with the host bone interface and reduces loosening of the implant in the initial stage of implantation [10,11,12]. Thus, porous Ta has broad application prospects in the field of bone defect repair.

The cellular structure of porous Ta implants has a direct impact on its mechanical properties, and also plays a very important role in the bonding between the implant and the host bone. The effects of different cellular microstructure characteristics of porous Ta implants on the mechanical properties, biological properties, bone integration ability, and bone formation need to be further studied. Huang et al. chose diamond as the cell structure, and found that the microstructure can be selected according to the biological performance requirements or mechanical requirements of the application environments [13]. Gao et al. studied the compressive mechanical behavior and failure mechanism of porous Ta, and found that the structural failure sites were mostly located in vertical struts and their connections [14]. Wang et al. designed and manufactured a new three-dimensional, multi-scale interconnected porous Ta scaffold, and biological experiments in vivo and in vitro showed that the microstructure characteristics of the new porous Ta scaffold were similar to bone trabeculae, and it had potential for bone tissue engineering applications [15]. Wauthle et al. tested the mechanical properties of porous Ta scaffolds with 80% porosity prepared with a regular dodecahedral cell structure, and the results showed that it was conducive to reducing the stress shielding effect within the range of human canceller bone [1]. Yang et al. used LPBF technology to fabricate porous Ta structures, and their microstructure showed a rough surface, with promising application prospects in bone filling and reconstruction [16]. It should be noted that Ta-related nanostructured materials can be obtained by simpler process methods, such as electro-chemical anodizing [17]. Additionally, nano-structured Ta oxide is more corrosion-resistant than pure metal, with great value for application in the medical field. In addition, the morphological characteristics of internal channels in porous structures also affect the mechanical properties, bone integration, and bone growth ability of implants [18,19,20]. Thus, the excellent bone integration properties of porous Ta scaffolds are closely related to their micro-mechanical properties and permeability properties. However, the existing microstructure design of porous Ta scaffolds follows the design scheme of Ti alloy, and the influence of microstructure on its mechanical properties and permeability is still unclear, which could lead to a bottleneck of porous Ta applications. Due to the superior mechanical and biological properties of porous Ta, previous studies on porous optimization schemes based on porous Ti cannot work well for porous Ta scaffolds. Therefore, it is urgent to design a microstructure optimization method suitable for porous Ta scaffolds.

In this paper, the influence of microstructures of porous Ta with diamond crystal cells on the mechanical properties and permeability was systematically investigated, and an optimized microstructure design was proposed. On this basis, the porous Ta structures were prepared by 3D printing and evaluated before and after microstructural optimization. This study could provide a reference for the optimal design of the cellular structure of clinical porous Ta implants.

## 2. Materials and Methods

### 2.1. Optimal Design Strategy of Porous Ta Microstructure

**Figure 1:**
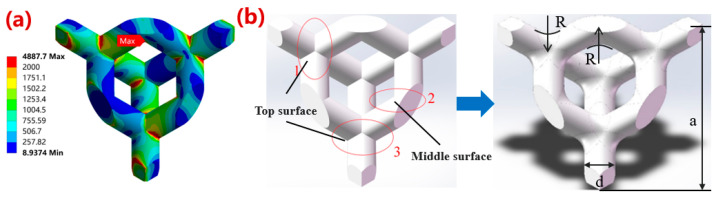
Schematic diagram of optimization of microstructural single cells. (**a**) Stress nephogram of a unit diamond cell structure; (**b**) optimized region division and unit cell fillet optimization design. The numbers 1 and 3 refer to the top corner optimization area, and the number 2 refers to the middle corner optimization area. The letter R refers to the radius of the rounded corner, and the letters a and d refer to the height and diameter of the cell, respectively.

In this study, a diamond crystal cell was selected for porous Ta structures. The mechanical properties of the structures were simulated by ANSYS v2022 software. According to the analysis of a stress cloud diagram of the unicellular structure (Figure 1a), the stress concentration effect mainly occurred at the intersection of two struts on the top surface (Figure 1b, region 2), followed by the intersection of the structs on the middle surface of the unicellular structure (Figure 1b, regions 1 and 3). Based on this, the single-cell structure optimization areas were divided as shown in Figure 1b. Due to the isotropy of the structure, regions 1 and 3 were regarded as the top surface, and region 2 as the middle surface. The forming of the single-cell struct was achieved by rotating along the central axis. This research investigated the optimization design parameters with variable fillets on the top and middle surfaces. The maximum stress, coefficient of variation, kurtosis, and skewness were used as criteria to analyze and evaluate the mechanical properties. Different optimization schemes are shown in Table 1. In order to ensure the unity of the structures, the porosities of the porous structures were kept at 80%, which is commonly applied in bioimplants, by changing the strut diameters.

### 2.2. Simulation of Mechanical Properties and Permeability for Optimization

#### 2.2.1. Mechanical Properties Simulation

**Figure 2:**
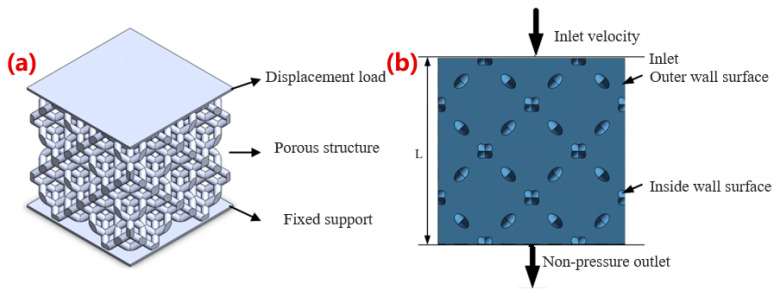
Simulation diagram of porous structure performance. (**a**) Loading conditions for mechanical simulation of porous structures; (**b**) fluid domain model and boundary condition setting of porous structures.

A static mechanical simulation was carried out to study the influence of different optimization parameters on the mechanical properties of the porous Ta structures. Two substrates were added on the top and bottom of the porous structures to match the stress environment during the compression experiments. The properties of the substrates were set as rigid bodies, the thickness of the substrates was 0.2 mm, and the side length was equal to the length of the porous structures. A schematic diagram of the overall structures is shown in Figure 2a. Considering the irregular structures in this study, the mesh type selected was a tetrahedral mesh. A downward displacement load was applied to the upper substrate as 1% of the total support height with a fixed lower substrate. The equivalent elastic modulus and stress concentration effect were analyzed. The stress concentration effect can be directly observed through the stress–strain cloud map, and the equivalent elastic modulus was mainly solved by the following formula [21]:

$$E=\frac{\sigma }{ԑ}=\frac{R_{Y}\cdot L}{∆L\cdot A}$$

R_Y_—axial branch reaction of fixed constraint (N); A—nominal contact area of porous structure (mm^2^); ∆L—deformation in the loading direction of the support (mm); L—length of support (mm).

#### 2.2.2. Permeability Simulation

A fluid simulation was carried out to study the influence of optimization parameters on the permeability of the porous structures. The fluid would enter from the upper surface, flow through the inner and outer walls in the microstructure, and exit from the lower surface as shown in Figure 2b. The liquid selected was water to simulate the flow trend of blood in the human body, with a density of 1000 kg/m^3^, a dynamic viscosity of 0.001 Pa/s, an inlet flow rate of 0.001 m/s, an and outlet pressure of 0 Pa. The permeability of porous structures was calculated according to Darcy’s law [22]:

$$k=\frac{\nu ul}{∆p}$$

k—permeability of porous structure (m); ν—inlet velocity of porous structure (m·s^−1^); l—total height of porous structure model (m); ∆p—the pressure gradient of the fluid domain, that is, the pressure difference between the upper and lower surfaces of the fluid domain (Pa).

Wall shear stress (WSS) is defined as the force per unit area exerted by the wall upward of the fluid in the local tangent plane [23]. Previous studies showed that it is conducive to cell growth when the wall shear stress ranges from 5 × 10^−5^ Pa to 2.5 × 10^−2^ Pa [24]. Otherwise, it would inhibit cell growth. So, the proportion of suitable areas for cell survival was also calculated as the ratio of the area with appropriate shear stress to the whole area.

### 2.3. Manufacturing of Porous Ta

**Figure 3:**
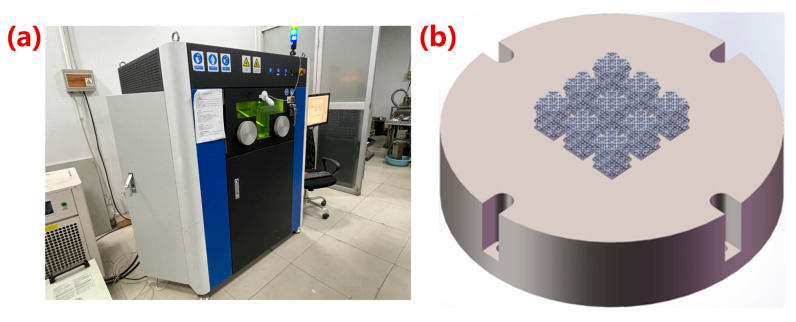
Porous Ta manufacturing platform. (**a**) LPBF additive manufacturing system; (**b**) sample preparation diagram.

The manufacturing equipment in this study was an LPBF (Laser Powder Bed Fusion) additive manufacturing system independently developed by Xi’an Jiaotong University (Figure 3a). The apparatus was equipped with a YLR-500-WC fiber laser with a rated power of 500 W, a spot diameter of 45 μm, and a wavelength of 1070.94 nm. The spherical Ta powder was provided by Suzhou Yaoyi New Material Technology Co., Ltd., Suzhou, China. The purity was 99.99% and the particle size was 15–53 μm. Porous structure models of 12 × 12 × 12 mm^3^ before and after optimization were established for preparation (Figure 3b). After the samples was fabricated, they were firstly sandblasted to remove the unmelted powder adhering to the surface of the structs, and then, cut from the substrate using a wire cutting machine (WGM4, Bossage CNC Equipment Manufacturing Co., Ltd., Suzhou, China). Finally, the samples were cleaned in an ultrasonic cleaning machine with anhydrous ethanol for 1 h. The optimal process parameters (P: 150 W, v: 270 mm/s, t: 0.05 mm, h: 0.07 mm) were selected to manufacture the structures with 80% porosity [25]. A total of 10 samples were prepared with 5 samples with and without optimization, respectively.

### 2.4. Evaluation of Properties of Porous Ta Optimization

#### 2.4.1. Microstructure Observation

A scanning electron microscope (Gemini500, ZEISS Microscopy, Jena, Germany) was used to observe the microstructure of the porous structures, and Image J software was used to measure the diameters of the struts. Ten different positions in each sample were selected for measurements of the average values. The porosities of porous Ta samples after post-treatment were measured using the weighing method.

#### 2.4.2. Mechanics Performance Testing

The samples were compressed along the forming and horizontal directions at a constant loading rate of 1 mm/min until failure by an electronic universal material testing machine (CMT4304, Max. 35 kN, Xian Letry Testing Machinese Co., Ltd., Xi’an, China). The compression experiment was set according to the national standard ISO 13314:2011 [26] (mechanical testing of metals–ductility testing–compression test for porous and cellular metals). The strain values of the porous structures during compression were measured by a force–displacement (stress–strain) sensor on the test machine, and the elastic modulus and yield strength of the porous structures were calculated according to the stress–strain curves.

#### 2.4.3. Permeability Testing

In the permeability tests, sealing tapes were used to seal four sides of the porous Ta samples in the fixture to ensure that the sample would not leak during the test. The flow rate at the inlet of the pipe was controlled to keep the height of the horizontal plane unchanged. During the test, the time required for each sample to discharge 500 mL water was measured. The test was repeated 5 times for each sample to calculate the average penetrating time.

### 2.5. Statistical Analysis Method

The statistical analysis was performed by one-way ANOVA and Student’s *t*-test using SPSS 25.0 (IBM Corporation, Armonk, NY, USA). The maximum stress, coefficient of variation, kurtosis, and skewness were calculated as criteria to evaluate the mechanical properties. The maximum stress reflected the influence of different optimization schemes on the stress concentration effect. The coefficient of variation represented the stress distribution in the analysis area. The skewness demonstrated the asymmetry of the mechanical properties data. All skewness values greater than 0 indicated that the stress distribution is a positive skewness distribution, that is, arithmetic mean > median > mode. The kurtosis represented the steepness of the data distribution. The kurtosis value of data that completely follow a normal distribution is 0. When the kurtosis value is greater than 0, it indicates that the data distribution is in a steep peak state and has a heavier tail than the normal distribution; on the contrary, when the kurtosis value is less than 0, it indicates that the distribution has a lighter tail and belongs to the flat peak state. The absolute value of kurtosis indicates how close the distribution is to the normal distribution [27,28,29].

## 3. Results and Discussion

### 3.1. Simulation Analysis Results

#### 3.1.1. Simulation Results of Mechanical Properties

**Figure 4:**
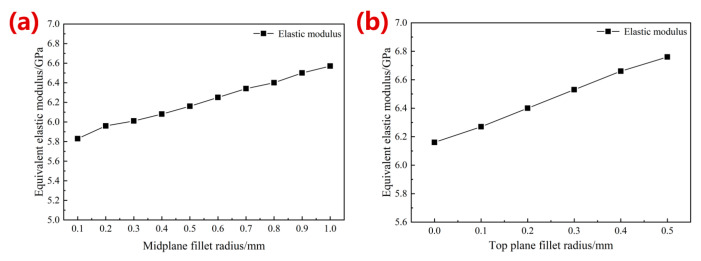
Simulation results of equivalent elastic modulus of different microstructures with an increase in the middle-plane fillet radius (**a**) and top-plane fillet radius (**b**).

**Figure 5:**
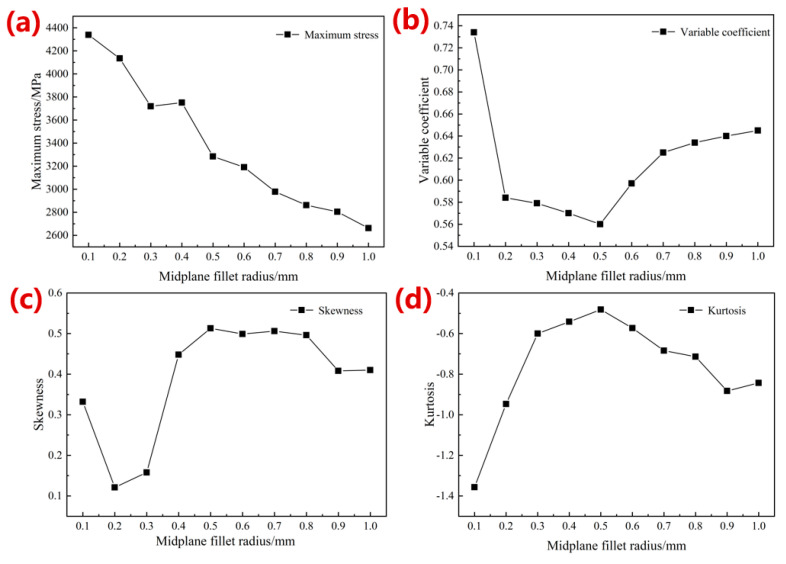
Simulation results of (**a**) maximum stress, (**b**) coefficient, (**c**) skewness change curve, and (**d**) kurtosis during optimization for the radius of middle-plane fillets.

Figure 4a shows the simulation results of the equivalent elastic modulus after optimization with different radii of the middle-plane fillets. With an increase in the radius, the elastic modulus gradually grew from 5.83 GPa to 6.57 GPa, about 14.66% higher than the original modulus (5.73 GPa). The maximum stress, coefficient of variation, skewness, and kurtosis change curves after optimization are shown in Figure 5. The maximum stress gradually decreased with an increase in the radius of middle-plane fillets, from 4338.80 MPa to 2662.30 MPa, with a maximum reduction of 50.66% compared with that of the original porous structure (5395.70 MPa). The coefficient of variation of all optimized structures was greater than 0, indicating that the stress distribution was not uniform. The structure with a radius of 0.50 mm showed the smallest coefficient of variation (0.56), indicating a relatively more uniform stress distribution in the structures. As shown in Figure 5c, the skewness value was 0.51 with a 0.50 mm radius of the middle-plane fillets, indicating that the positive skewness of the structure was more obvious at this time. Most of the stress values were small, demonstrating that the stress concentration effect was significantly improved. The kurtosis values of all structures were less than 0, demonstrating that the stress distribution of the optimized structures belonged to the flat peak state, with a minimum value of −1.36 when the radius was 0.10 mm, and a maximum value of −0.48 when the radius equaled 0.50 mm. The absolute value of kurtosis reached its smallest when the optimization radius was 0.50 mm, with a more uniform stress distribution, as shown in Figure 5 Thus, this study selected a midplane fillet radius of 0.50 mm.

**Figure 6:**
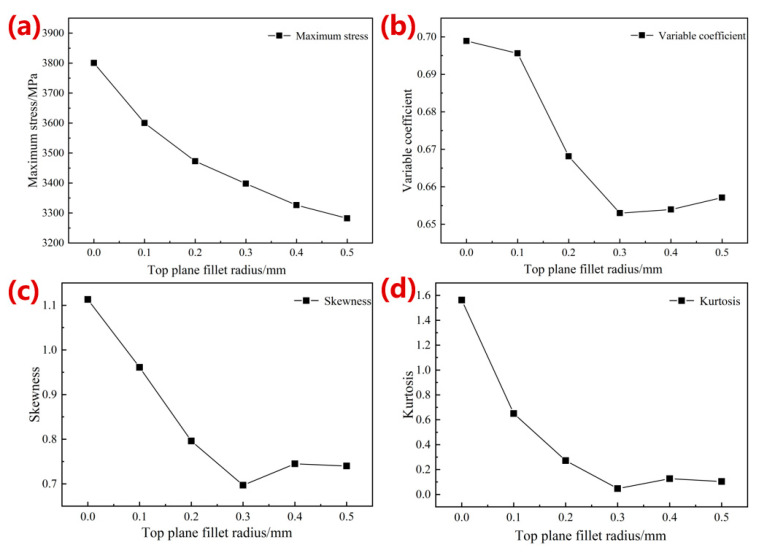
Simulation results of (**a**) maximum stress, (**b**) coefficient, (**c**) skewness change curve, and (**d**) kurtosis with the increase in the radius of top-plane fillets.

Figure 4b shows the simulation results of the equivalent elastic modulus with radius optimization of the top-plane fillets. The modulus of the optimized structure was further improved by 9.74% with an increase in fillet radius, from 6.16 GPa to 6.76 Gpa. The maximum stress, coefficient of variation, skewness, and kurtosis curves of the structures after the top fillet optimization are shown in Figure 6. All skewness values greater than 0 indicated that the stress distribution after the top fillet optimization was also a positive skewness distribution; all kurtosis values greater than 0 revealed that the stress distribution curve was a steep peak state. And the smaller the absolute value of kurtosis value was, the more concentrated the stress distribution was around the arithmetic mean value. With the increase in the optimized radius, the maximum stress decreased gradually, and a minimum value of 3282.10 MPa was obtained when the radius was 0.5 mm, with a decrease of 13.64%. The coefficient of variation, kurtosis, and skewness obtained the minimum values at a radius of 0.30 mm, which were 0.65, 0.70, and 0.05, respectively. With an optimization radius of 0.3 mm, the small coefficient of variation indicated a more uniform stress distribution, and the minimum skewness and kurtosis values demonstrated a smaller stress distribution range.

**Figure 7:**
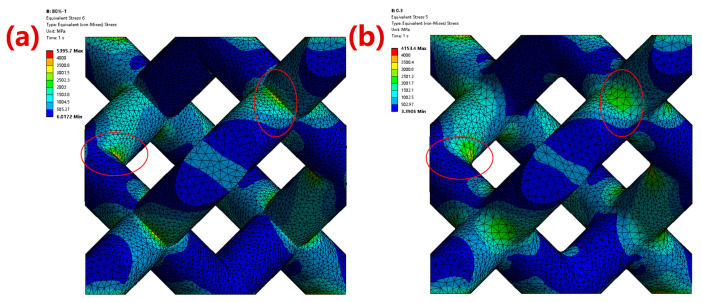
Stress nephogram comparison between (**a**) the original cell structure and (**b**) the optimized cell structure. The red circle areas represent the rounded corner optimization area.

Combined with the simulation results of fillet optimization of the midplane and top plane, the equivalent elastic modulus of the 80%-porosity cell structure increased by 13.96% from 5.73 GPa to 6.53 GPa (midplane fillet radius: 0.5 mm; top-plane fillet radius: 0.3 mm). The maximum stress was reduced by 39.17% from 5395.70 MPa to 3282.10 MPa, the stress distribution range was smaller and more uniform, and the stress concentration effect was significantly improved, as shown in Figure 7.

#### 3.1.2. Simulation Results of Permeability Performance

**Figure 8:**
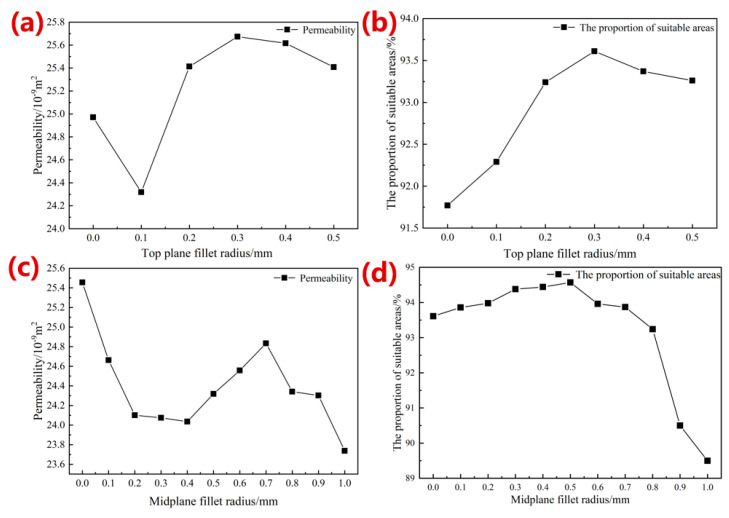
Simulation results of permeability and the proportion of biologically suitable areas with radius increase of top-plane fillets (**a**,**b**) and midplane fillets (**c**,**d**), respectively.

Figure 8a,b show the results of the permeability and the proportion of suitable areas for cell survival with the radius optimization of top-plane fillets. As the radius became larger, the permeability of the porous structures decreased at the beginning, and then, increased. When the radius was 0.3 mm, the maximum permeability was 25.67 × 10^−9^ m^2^, with an increase of 2.81%. Similarly, the proportion of suitable areas for cell survival also reached the maximum value of 93.61% at this point (Figure 8b). Thus, the optimized results of the radius of the top-plane fillets based on permeability were consistent with the results from the mechanical properties, and a radius of 0.30 mm was selected for the top-plane fillet radius after optimization.

Figure 8c,d shows the permeability and the proportion of suitable areas for cell survival results with midplane fillet radius optimization. The proportion of the biologically suitable areas firstly decreased when the radius was less than 0.7 mm, and then, increased with the radius change. On the other hand, the proportion of suitable areas for cell survival remained stable when the radius was smaller than 0.7 mm, and reduced suddenly with a larger radius (Figure 8d). Therefore, this research chose a midplane fillet radius of 0.50 mm to keep consistence with the mechanical properties analysis.

**Figure 9:**
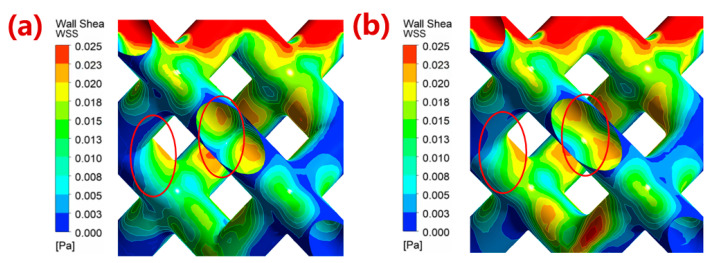
Comparison of WSS distribution cloud image between (**a**) original single cell structure and (**b**) cell structure with optimization of fillet radius (top-plane fillet radius: 0.3 mm, midplane fillet radius: 0.5 mm). The red circle areas represent the rounded corner optimization area.

Figure 9 shows the comparison of the WSS distribution of the single-cell structure before and after fillet radius optimization of the top plane and midplane, and the WSS range was set to 5.0 × 10^−5^ to 2.5 × 10^−2^ Pa. It can be seen that the WSS distribution of the optimized structure was more uniform, which is more conducive to cell adhesion. Figure 9b compared the WSS distribution cloud map before and after the optimization of the fillets. It can be seen from the figure that the structure optimization effectively improved the phenomenon of the sudden change at the angle of the struct, and made the WSS distribution more uniform. According to the simulation results of different fillet radius designs, the permeability and proportion of the biologically suitable areas of 80%-porosity porous structures with optimized fillet radii of 0.50 mm on the top plane and 0.30 mm on the midplane are 24.318 × 10^−9^ m^2^ and 94.57%, respectively, which is consistent with the permeability of human bone structure.

### 3.2. Porous Model after Microstructure Optimization for Manufacturing

**Figure 10:**
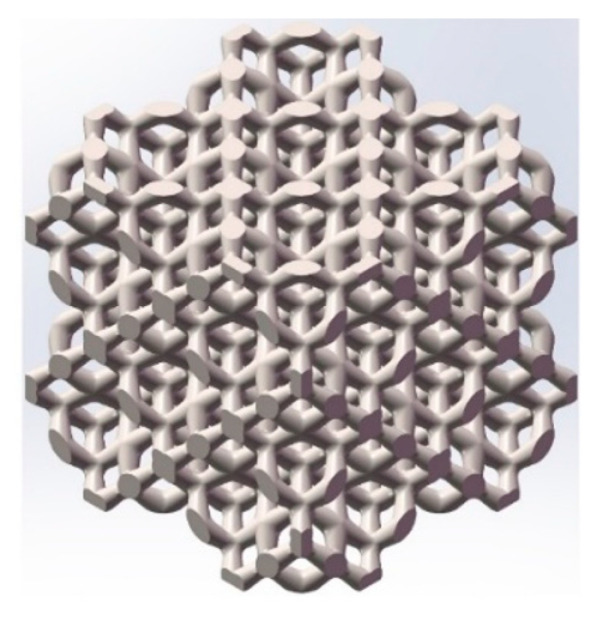
Porous structure with fillet optimization.

A suitable porous microstructure design can not only improve the carrying capacity and long-term stability of porous structures and reduce the stress concentration effect inside the structure, but also improve the permeability of porous structures, which is more conducive to the transport of nutrients and cell adhesion in the porous structure after implantation. Based on the results of the microstructure optimization of mechanical properties and permeability properties, a porous structure with fillet radius optimization was established (Figure 10). The radius of fillets of the midplane was 0.50 mm, and the radius of the top surface fillet was 0.30 mm.

### 3.3. Morphology of Porous Ta with Optimized Microstructure

**Figure 11:**
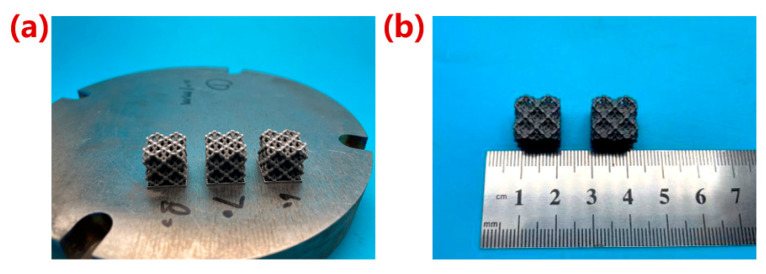
Macroscopic photographs of porous Ta scaffold samples. (**a**) Macroscopic photographs; (**b**) macroscopic geometry size [25].

**Figure 12:**
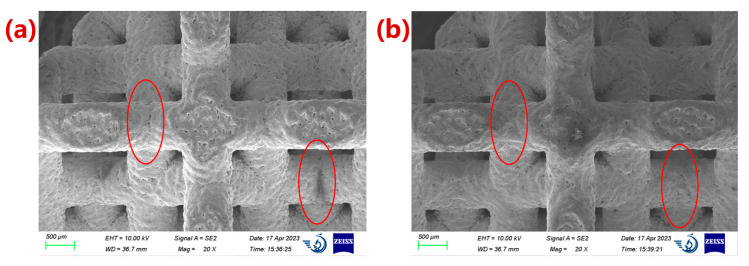
Electron microscopic photos of microstructure of porous Ta. (**a**) The original microstructure; (**b**) the fillet-optimized microstructure. The red circle areas represent the rounded corner optimization area.

The prepared porous Ta samples are shown in Figure 11 [25]. Compared with the microstructure of the un-optimized structure, it can be seen that the optimization of the fillets could effectively improve the non-uniformity of the connection transition between the struts (Figure 12), which helped reduce the stress concentration effect inside the structure and improve its mechanical properties. The results showed that the strut diameters of the porous structure after optimization in the horizontal direction and the forming direction were 0.86 ± 0.01 mm and 0.80 ± 0.01 mm, respectively. The difference in the strut diameter before and after the optimization was not significant. Due to the existence of fabrication error, the porosities of the porous structures before and after optimization were still smaller than the design value of 80%.

### 3.4. Mechanical Test Results of Porous Ta with Optimized Microstructure

**Figure 13:**
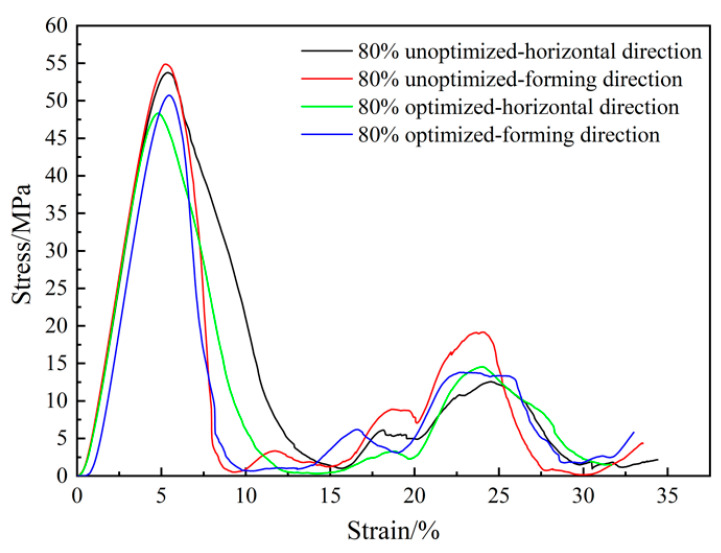
Compressive stress–strain curve results of porous Ta with and without fillet optimization scheme.

The compressive stress–strain curves of the fillet-optimized structure are shown in Figure 13. The elastic modulus and yield strength of different porous structures were measured, and the results are shown in Table 2. The results showed that the optimized design of a porous structure can effectively improve the yield strength, while the elastic modulus increased slightly after optimization. This might be due to the fact that the optimized design could improve the stress concentration effect during loading with a relatively smooth transition between the struts of porous structures.

### 3.5. Permeability Test Results of Porous Ta with Optimized Microstructure

The results demonstrated that the permeability of the 80%-porosity porous structure increased by 8.25% from 6.63 ± 0.16 × 10^−9^ m^2^ to 7.18 ± 0.18 × 10^−9^ m^2^ after optimization. This might be due to the fact that the transition of the struts in the porous structure after the fillet optimization was more uniform, and the permeability was increased by improving the pore distribution inside the structures. Both of the permeability test results and the simulation results show the same change trend, indicating that the established permeability calculation model had good reliability. The test results were smaller than the simulation values. This may be result from two reasons: the strut diameter of the fabricated porous structure was too large, causing the shape of the fluid domain to be different from the simulation model; the manufactured struts had a certain degree of roughness, and some liquid droplets would be adsorbed on the struts when the liquid flowed through the surfaces of the struts, thus affecting the permeability. Therefore, fillet optimization can improve the mechanical properties and permeability of porous structures, which is consistent with the simulation results.

## 4. Conclusions

This paper created a fillet optimization design with a porous cell structure, obtained the optimal design parameters through the simulation of mechanical and penetration properties, and verified the results experimentally using porous Ta. We determined the following based on the above simulation and experimental analysis results:

(1) The optimized parameters of the porous diamond structure are as follows: the fillet radius of the top plane is 0.50 mm, and the radius of the midplane fillet is 0.30 mm. The equivalent elastic modulus of the 80%-porosity cell structure is increased by 13.96%, the maximum stress is reduced by 39.17%, and the proportion of the suitable area for cell growth is increased by 1.15%.
(2) The elastic modulus in the forming and horizontal directions of the porous Ta before and after fillet optimization are increased from 1.298 ± 0.006 GPa and 1.352 ± 0.007 GPa to 1.328 ± 0.002 GPa and 1.384 ± 0.006 GPa, representing increases of 2.31% and 2.37%, respectively. At the same time, the permeability is increased by 8.25% from 6.63 ± 0.16 × 10^−9^ m^2^ to 7.18 ± 0.18 × 10^−9^ m^2^.
(3) The yield strength of the optimized porous Ta samples prepared by LPBF has a relatively obvious increase of 10.39%. This might be due to the fact that the porous Ta after the fillet optimization of can effectively reduce the stress concentration effect.

Compared with previous studies on the structural design optimization of porous scaffolds [13,14,16,18,19,20], the microstructure optimization of porous Ta in this study can maximize the mechanical properties and permeability based on the excellent mechanical and biological properties of porous Ta. Although this fillet optimization scheme can also be applied to porous Ti scaffolds, the porous Ta scaffold could demonstrate superior performance with better innate properties as bioimplants. Furthermore, the optimization method in this study combined with the mechanical properties of porous Ta can enable the preparation of porous structures with higher porosity and permeability that meet the mechanical requirements of bioimplants, which are incomparable to porous Ti scaffolds. This has important significance for the research and application of porous Ta scaffolds with high porosity.

## Figures and Tables

**Table 1:** Single-cell structure optimization design parameters.

Parameters	Radius
Middle-plane fillets	0.1 mm~1.0 mm (interval 0.1 mm)
Top-plane fillets	0.1 mm~0.5 mm (interval 0.1 mm)

**Table 2:** Test results of mechanical properties between the porous Ta with original and optimized structures.

Structure Type	Elasticity Modulus (GPa)		Yield Strength (MPa)	
	Horizontal Direction	Forming Direction	Horizontal Direction	Forming Direction
Original structure	1.352 ± 0.007	1.298 ± 0.006	53.981 ± 0.124	49.771 ± 0.091
Optimized structure	1.384 ± 0.053	1.328 ± 0.009	59.590 ± 0.053	54.226 ± 0.010

## Data Availability

The data presented in this study are available on request from the corresponding author.
